# Clinical efficacy of acupuncture therapy combined with core muscle exercises in treating patients with chronic nonspecific low back pain: a systematic review and meta-analysis of randomized controlled trials

**DOI:** 10.3389/fmed.2024.1372748

**Published:** 2024-04-04

**Authors:** Xia Li, Guohua Zhai, Hongkai Zhang, Xuefei Li, Mingqi Wu, Sidi Zhang, Jiawen Cui, Zhanying Tang, Zhijun Hu

**Affiliations:** ^1^Longhua Hospital, Shanghai University of Traditional Chinese Medicine, Shanghai, China; ^2^Fenglin Community Health Service Centre, Shanghai, China; ^3^Longhua Clinical Medical College of Shanghai University of Traditional Chinese Medicine, Shanghai, China; ^4^School of Rehabilitation Medicine, Shanghai University of Traditional Chinese Medicine, Shanghai, China; ^5^Jinshan District Hospital of Integrated Chinese and Western Medicine, Shanghai, China

**Keywords:** acupuncture therapy, core muscle exercises, chronic nonspecific low back pain, pain, clinical efficacy, dysfunction

## Abstract

**Introduction:**

This meta-analysis aimed to determine the clinical efficacy of acupuncture combined with core muscle exercises on pain and functional status in patients with chronic nonspecific low back pain.

**Methods:**

This study followed the Preferred Reporting Items for Systematic Reviews and meta-analysis criteria for systematic reviews and meta-analyses. Randomized controlled trials published till November 2023 were searched in PubMed, Web of Science, Cochrane, Embase, China National Knowledge Infrastructure, Chinese Biomedical Literature, and Wanfang databases. The search strategy was related to disease type, intervention, and control measures and was structured around the search terms “low back pain,” “acupuncture therapy,” and “exercise.” Two reviewers applied inclusion and exclusion criteria. Sensitivity and fixed effects analyses were performed to determine the primary outcomes.

**Results:**

We included 11 randomized controlled trials (*n* = 727) on acupuncture combined with core muscle exercises in patients with chronic nonspecific low back pain. Compared with controls, clinical efficacy was significant, with improvements in pain scores (visual analog pain scale and numerical rating scale) and Oswestry Disability Index in the intervention group.

**Discussion:**

Acupuncture therapy combined with core muscle exercises improved pain and functional status in patients with chronic nonspecific low back pain, with favorable clinical outcomes compared with single-core muscle training. Multicenter large-sample trials are required to obtain more reliable conclusions.

## 1 Introduction

Low back pain (LBP) and acute and chronic pain in the posterior of the lumbar gluteal region between the 12th rib margin and the subgluteal fold are common clinical conditions classified into two major categories: idiosyncratic (caused by a specific etiology of spinal or non-spinal origin) and non-idiosyncratic (1). Nonspecific low back pain (NLBP) accounts for over 85% of LBP, and its diagnosis requires excluding specific pathological causes. The disease progresses to chronicity over 3 months of illness, primarily manifesting as pain and disability (2–4). In the Chinese context, “disability” refers to conditions that can cause short- or long-term health losses (5). According to epidemiological surveys, the number of people with chronic nonspecific low back pain (CNLBP) worldwide is approximately 568.4 million, with an average prevalence of approximately 18.3% and a lifetime prevalence reaching 47%. LBP has become the primary cause of years lived with disability worldwide, causing extensive medical expenditure, social burden, and productivity loss to families, communities, and countries (2, 6, 7).

Non-pharmacological interventions dominate the first-line treatment for CNLBP (8, 9). Regular exercise programs can significantly improve pain, function, posture, health status, and quality of life (8, 10, 11). The guidelines recommend exercises that activate the multifidus and transversus abdominis muscles, the primary core muscles of the lumbar spine that maintain lumbar spine stability, to improve pain and disability in patients with CNLBP (12, 13). Acupuncture is one of the most important means of traditional disease prevention and treatment in China. A large number of fundamental and clinical studies have confirmed that acupuncture has the therapeutic effects of correcting endocrine metabolism disorders, relieving pain, regulating mental health, and improving the quality of life, and that it plays an important role in neurology, connective tissue pathology, mental health, and other related fields (14).

High-quality evidence strongly recommends that patients with CNLBP should engage in physical exercise whenever possible; however, the quality of the evidence recommending acupuncture therapy is inconsistent (2–4, 9, 15). A systematic review published in 2022 addressed core stability exercises versus conventional exercise for chronic LBP, using meta-analysis to include 14 relevant studies, concluding that core stability exercises were superior to conventional exercise regarding short-term pain relief and improvement in functional disability (16). Another 2023 systematic review (comprising meta-analyses) reported acupuncture as an alternative or complementary treatment to conventional treatment for CNLBP and had six subgroups where acupuncture alone or combined with conventional treatment acupuncture were compared with conventional treatments (pharmacological, non-pharmacological, and combined pharmacological and non-pharmacological). Combined acupuncture and non-drug treatments reportedly further improve pain and disability; however, the quality of evidence is low, and only one randomized controlled trial (RCT) with exercise control was included (17). No relevant systematic review has demonstrated the therapeutic effects of acupuncture combined with core exercise programs. In the light of the above, this systematic review and meta-analysis aimed to assess the effectiveness of acupuncture combined with core muscle exercises in treating CNLBP, especially in improving patients’ pain and disability.

## 2 Information sources and search strategies

Reference data were searched using the following electronic databases: PubMed, web of science, Cochrane, Embase, China national knowledge infrastructure (CNKI), Chinese biomedical literature, and Wanfang. We systematically searched the above databases for articles published till November 23, 2023, without language restrictions.

The search criteria were based on participants, intervention, comparison, outcome, time, and study design (PICOTS), and the search strategy was correlated with disease types, intervention, and control measures and was structured around the search terms “low back pain,” “acupuncture therapy,” and “exercise.” Subject terms, their synonymous free words, and qualifiers were used to improve search sensitivity: (“Low Back Pain” OR “Back Pain, Low” OR “Back Pains, Low” OR “Low Back Pains” OR “Pain, Low Back” OR “Pains, Low Back” OR “Lumbago” OR “Lower Back Pain” OR “Back Pain, Lower” OR “Back Pains, Lower” OR “Lower Back Pains” OR “Pain, Lower Back” OR “Pains, Lower Back” OR “Low Back Ache” OR “Ache, Low Back” OR “Aches, Low Back” OR “Back Ache, Low” OR “Back Aches, Low” OR “Low Back Aches” OR “Low Backache” OR “Backache, Low” OR “Backaches, Low” OR “Low Backaches” OR “Low Back Pain, Postural” OR “Postural Low Back Pain” OR “Low Back Pain, Posterior Compartment” OR “Low Back Pain, Recurrent” OR “Recurrent Low Back Pain” OR “Low Back Pain, Mechanical” OR “Mechanical Low Back Pain”) AND (“Acupuncture Therapy” OR “Acupuncture Treatment” OR “Acupuncture Treatments” OR “Treatment, Acupuncture” OR “Therapy, Acupuncture” OR “Pharmacoacupuncture Treatment” OR “Treatment, Pharmacoacupuncture” OR “Pharmacoacupuncture Therapy” OR “Therapy, Pharmacoacupuncture” OR “Acupotomies” OR “Acupotomy”) AND (“Exercise” OR “Exercises” OR “Physical Activity” OR “Activities, Physical” OR “Activity, Physical” OR “Physical Activities” OR “Exercise, Physical” OR “Exercises, Physical” OR “Physical Exercise” OR “Physical Exercises” OR “Acute Exercise” OR “Acute Exercises” OR “Exercise, Acute” OR “Exercises, Acute” OR “Exercise, Isometric” OR “Exercises, Isometric” OR “Isometric Exercises” OR “Isometric Exercise” OR “Exercise, Aerobic” OR “Aerobic Exercise” OR “Aerobic Exercises” OR “Exercises, Aerobic” OR “Exercise Training” OR “Exercise Trainings” OR “Training, Exercise” OR “Trainings, Exercise”) AND (“randomized controlled trial” OR “randomized” OR “placebo”). In PubMed, search results were limited to “randomized controlled trials.” The Supplementary File contains further search strategies. The first author (XL) screened the studies by title and abstract according to the inclusion and exclusion criteria. In addition, a manual search of the references and abstracts of all the included articles and previous relevant systematic reviews and meta-analyses was conducted. The Preferred Reporting Items for Systematic Reviews and meta-analyses guided this systematic review and meta-analysis (18).

### 2.1 Inclusion criteria

The inclusion criteria of the articles is RCTS published in the above seven authoritative electronic databases. RCTS need to cover the following research components: (1) participants’ inclusion criteria were limited to patients with CNLBP, defined as disease duration beyond 3 months; (2) The control groups underwent exercises targeting the core muscles; (3) the intervention groups involved the addition of acupuncture therapy to the control group that contained general acupuncture (manual acupuncture), electroacupuncture, needle-knife, and fire-needle; (4) the outcomes were pain, disability, and clinical outcomes of the patients. using measures including the visual analog pain scale (VAS), numerical rating scale (NRS), oswestry disability index (ODI), and clinical effectiveness.

### 2.2 Exclusion criteria

We excluded studies with the following characteristics: (1) Unavailability of full text and/or incomplete data; (2) LBP attributable to a specific pathology (including pelvic or urinary tract infections, tumors, renal disease, osteoporosis, lumbar spine lesions, inflammatory disorders, and neurogenic syndromes); (3) Studies where acupuncture was applied to a specific “microsystem” (e.g., scalp, ear, eye, or buccal needling); (4) Forms of acupuncture combined with moxibustion or medication, such as warm needling, acupoint injections, or hydroentanglement; (5) The inclusion of two or more acupuncture therapies in the observational group; (6) Exercise that does not target the core musculature; (7) The use of pharmacological treatments in the study.

### 2.3 Study selection and data extraction

Two researchers (GZ and HZ) independently assessed the potentially relevant articles after reading the full text for final inclusion. Disagreements were discussed with other authors, and a third researcher (ZT) resolved differences. The information collected included the first author’s name, publication year, subject characteristics (mean age, sex, and disease duration), sample size, intervention (specific acupuncture therapy, exercise method, and duration of intervention), risk assessment, and outcome indicators.

### 2.4 Outcome measurement

In this systematic review and meta-analysis, the primary outcome was the pain score. The secondary outcomes were effectiveness and ODI scores.

### 2.5 Evaluation of research quality

Two researchers (XL and MW) independently assessed the methodological quality of each RCT using the Cochrane risk-of-bias assessment tool. Disagreements were resolved through discussions with a third investigator (ZH). The risk-of-bias assessment included random sequence generation, allocation concealment, blinding of participants and investigators, blinding of outcome assessment, completeness of outcome data, selective reporting, and other biases. All criteria were assessed equally at “low,” “unclear,” and “high” risk levels.

### 2.6 Data synthesis

All data analyses were performed using Review Manager version 5.3. Dichotomous outcomes were analyzed by calculating the relative risk for each trial, with the uncertainty of each outcome expressed as a 95% confidence interval (CI). When studies were assessed using the same scale, continuous outcomes were analyzed by calculating the mean difference of the 95% CI. When instruments were different, we used the standardized mean difference of the 95% CI. The statistical heterogeneity of the results of each study was evaluated using the Cochrane *Q*-test, and I^2^ values were quantified using the *Q*-test significance threshold *p* = 0.1 and *I*^2^ value (50%). The fixed-effects model was used when *I*^2^ was <50%, and heterogeneity was explored when *I*^2^ was >50%. The final results were presented as traditional meta-analytic forest plots.

### 2.7 Heterogeneity exploration and analysis

When there was statistical heterogeneity in the studies, we identified its potential causes through sensitivity analyses and used a random-effects model if it could not be eliminated and was <70%. Similarly, we prioritized sensitivity analyses, followed by subgroup analyses: classification of specific acupuncture therapies, patient age (less than or greater than 40 years), and disease duration (less than or greater than 12 months). However, we did not perform subgroup analyses because the final included studies were not significantly heterogeneous after sensitivity analyses to exclude some studies. We assessed possible publication bias by visually inspecting funnel plots (plots of effect estimates for each study versus sample size or standard error of the effect).

## 3 Results

### 3.1 Research options

**Figure 1:**
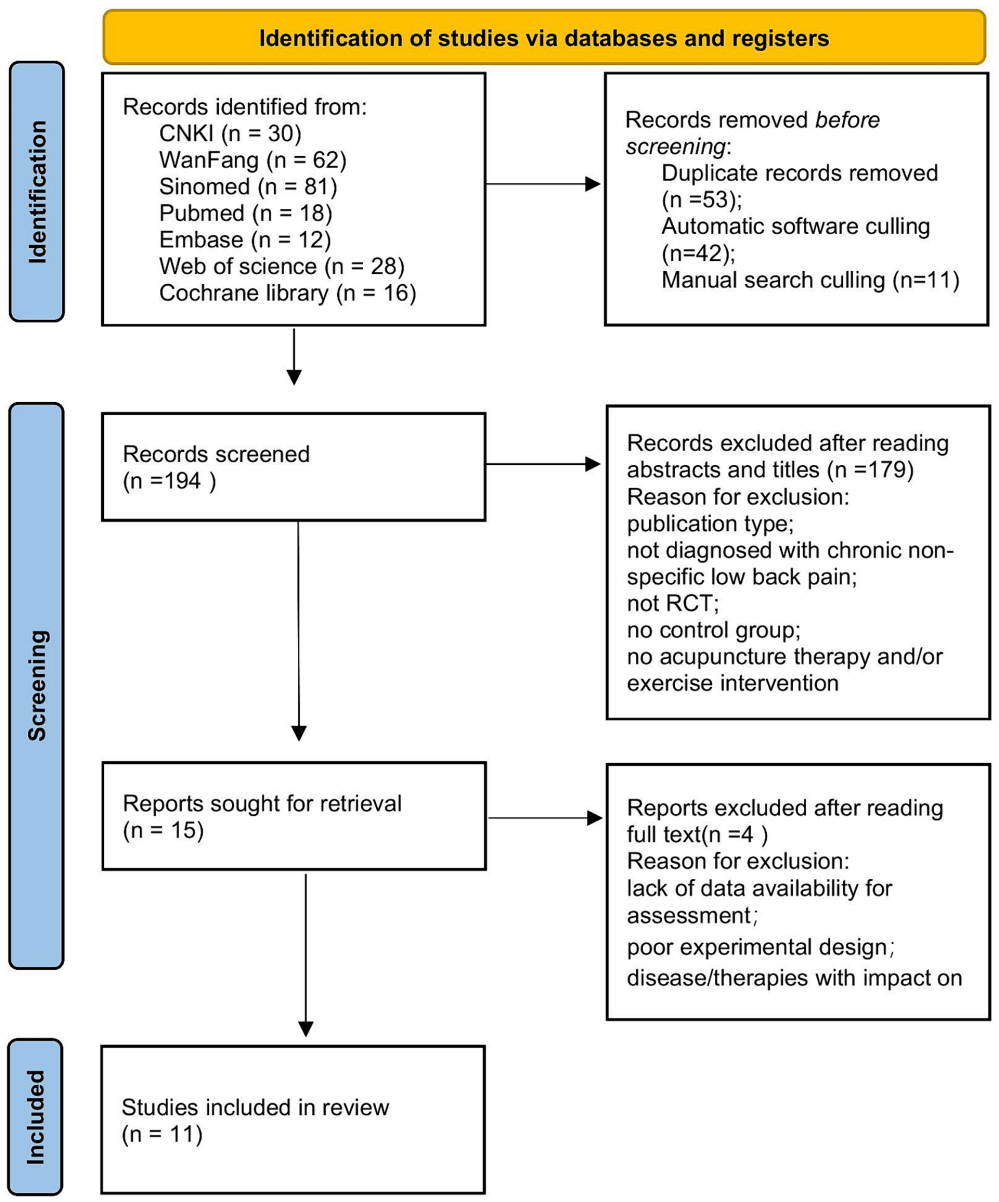
Preferred reporting items for systematic reviews and meta-analysis flow diagram.

We identified 247 studies from the selected databases, with 53 duplicate entries removed by document management software and manual searches. The remaining 194 studies were screened using titles and abstracts to exclude 179. The remaining 15 studies were assessed based on the inclusion and exclusion criteria described above. We selected 11 RCTs for meta-analysis (19–29). A flowchart is shown in Figure 1.

### 3.2 Study characteristics and interventions

The number of participants in the 11 RCTs was 727 (observation group, *n* = 364; control group, *n* = 363), with sample sizes in individual studies ranging from 26 (20) to 50 (19). The 11 trials included both sexes, with a predominantly young adult age profile and mean age fluctuations ranging from 26 years (23) to 55 years (20), and all included patients had NCLBP. In the 11 RCTs, the control group performed core muscle exercises, including suspension exercise training modalities; three were core muscle exercises performed by the treatment staff through the suspension training system (20, 23, 28), and the rest were self-exercised core muscle exercises guided by the treatment staff. The observation group received acupuncture therapy, while the control group received no specific treatment type. The intervention time and frequency of acupuncture therapy varied according to the specific type, ranging from 2 weeks (19, 22, 29) to 8 weeks (25), and the frequency of intervention from once weekly (25, 27, 29) to once daily (23, 26). For exercise therapy interventions, the duration ranged from 2 weeks (22) to 3 months (29), and the frequency of exercise ranged from once daily (20, 22, 23, 26–28) to once weekly (25). One RCT (23) specified only the number of interventions without frequency, and one RCT (28) did not explicitly explain the frequency of acupuncture therapy interventions. The details of the study characteristics and interventions are presented in Table 1.

**Table 1:** Characteristics of the included RCT studies.

First author, year	Sample size		Gender (M:F)		Mean age		Disease duration		Intervention duration		Exercise intervention	Control intervention	Outcome
	Exercisers	Controls	Exercisers	Controls	Exercisers	Controls	Exercisers	Controls	acupuncture therapy	Exercise therapy			
Li et al., 2020 (29)	30	30	1:1	17:13	48.1 ± 8.6	47.6 ± 7.2	8.67 ± 3.44	9.20 ± 4.10	1 session weekly, 2 weeks	4 sessions weekly, 3 months	Acupotomy + core stability training	core stability training	A;B;C
Liao, 2019 (24)	30	30	7:8	17:13	41.6 ± 5.01	42.13 ± 4.78	35.07 ± 1.59	37.47 ± 8.96	3 sessions weekly, 4 weeks	3 sessions weekly, 4 weeks	Traditional acupuncture + core muscle group training	core muscle group training	A;B;C
Han, 2017 (23)	30	30	13:17	2:3	26.71 ± 7.12	27.14 ± 6.65	10.22 ± 4.28	9.16 ± 3.52	1 session daily, 10 sessions	1 session dayly, 10 sessions	electroacupuncture + core strength training	core muscle group training	A
Liu et al., 2020 (22)	40	38	21:19	17:21	49.70 ± 7.13	47.05 ± 8.35	29.93 ± 13.94	26.89 ± 16.07	1 every other day, 2 weeks	1 session weekly, 2 weeks	floating needle therapy + core muscle group training	core muscle group training	A;C
Liu et al., 2021 (21)	42	42	10:11	3:4	36.64 ± 7.59	36.59 ± 7.82	30.27 ± 11.09	21.46 ± 2.49	5 sessions weekly, 4 weeks	5 sessions weekly, 4 weeks	electroacupuncture + core strength training	core strength training	A;B;C
Liu, 2012 (28)	20	20	1:1	4:3	43.28 ± 10.34	46.73 ± 11.58	66.78 ± 14.57	67.43 ± 13.55	/, 4 weeks	1 session weekly, 4 weeks	acupuncture + suspension core muscle training	suspension core muscle training	A
Jiang, 2021 (27)	40	40	23:17	11:9	49.50 ± 3.27	49.00 ± 3.28	8.00 ± 1.30	8.50 ± 1.28	1 session weekly, 3 weeks	1 session dayly, 20 days	bladed needle + core muscle group training	core muscle group training	A;B;C
Li et al., 2018 (26)	26	27	19:11	16:13	36.29 ± 4.61	36.95 ± 4.4	15.94 ± 5.08	14.98 ± 5.17	1 session dayly, 20 days	1 session dayly, 20 days	Tendon acupuncture + core stability training	core stability training	A;B
Huang, 2019 (25)	30	30	3:2	19:11	27.23 ± 4.13	27.44 ± 4.29	10.92 ± 3.29	10.36 ± 3.43	1 session weekly, 8 weeks	1 session weekly, 8 weeks	fire acupuncture + core muscle group training	core muscle group training	A;B;C
Wang and Zhu, 2020 (19)	50	50	14:11	27:23	36 ± 6	36 ± 6	8.7 ± 4.3	8.6 ± 4.1	6 sessions weekly, 2 weeks	3 sessions weekly, 4 weeks	Cangguitanxue acupuncture + suspension core muscle therapy	suspension core muscle training	A;B
Yeung et al., 2003 (20)	26	26	2:11	5:21	50.4 ± 16.3	55.6 ± 10.4	/	/	3 sessions weekly, 4 weeks	1 session weekly, 4 weeks	electroacupuncture + back muscles exercises	back muscles exercises	D

A, VAS scores; B, Efficiency; C, ODI scores; D, NRS scores.

The 11 RCTs involved assessing pain, low-back dysfunction, and treatment effects. 10 RCTs (19, 21–29) involved using VAS to assess overall pain, and one (20) involved using NRS to assess peak versus mean pain. Six RCTs (21, 22, 24, 25, 27, 29) involved using ODI to assess lumbar dysfunction, and the rest were conducted using The Aberdeen LBP scale (20), The Roland Morris disability questionnaire (26), and the Japanese Orthopedic Association Assessment Treatment score (23, 28). Three RCTs (23, 28, 29) were conducted using the Japanese Orthopedic Association Assessment Treatment score, and another (29) converted the Japanese Orthopedic Association Assessment Treatment score results to a percentage to evaluate the treatment effect. Seven RCTs (19, 21, 24–27, 29) involved using the efficiency rate to evaluate the clinical treatment, and the details of the remaining outcome indicators are presented in Table 1.

### 3.3 Methodological quality

**Figure 2:**
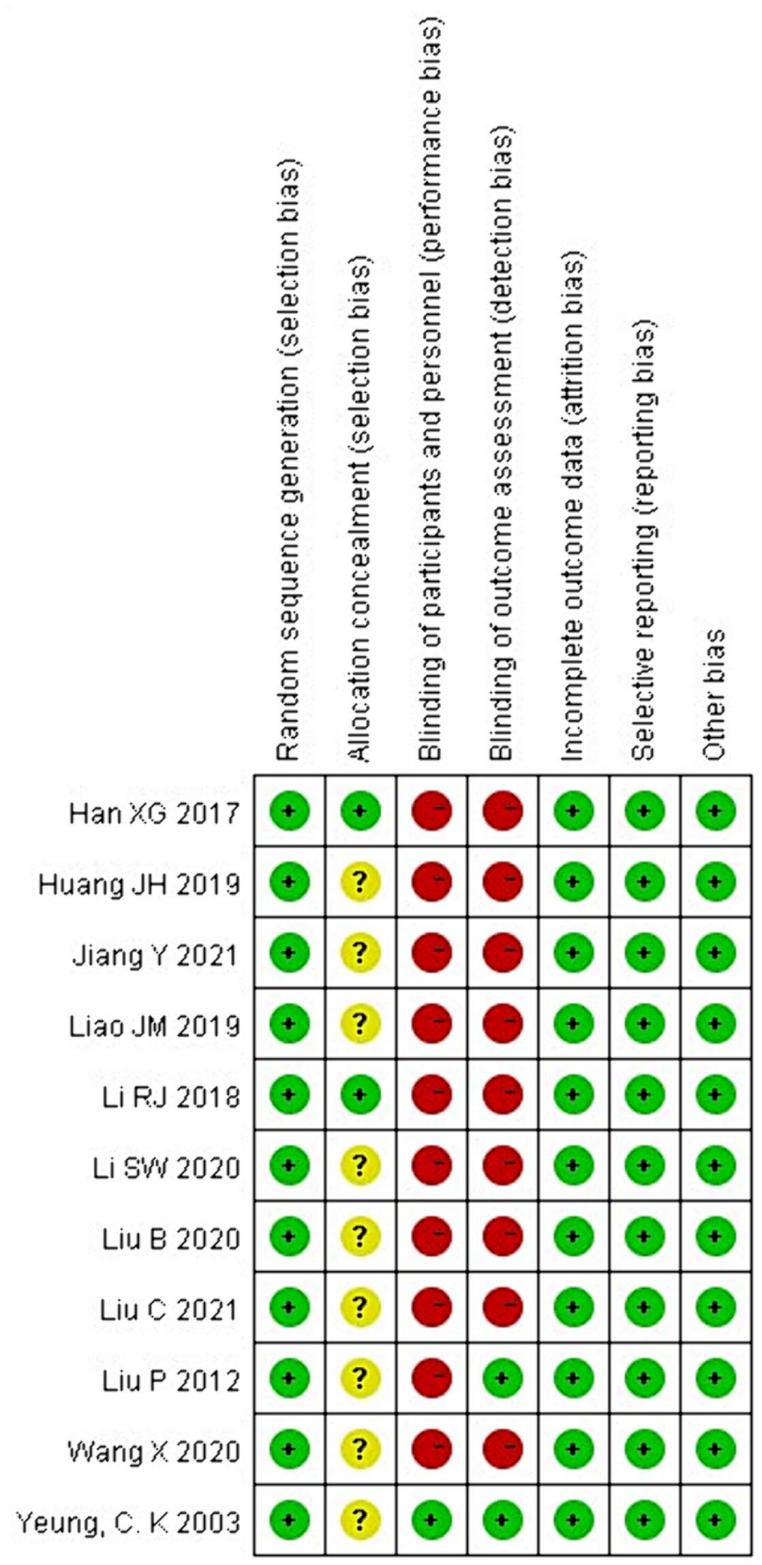
Risk of bias assessment summary of randomized control trials (RCTs).

**Figure 3:**
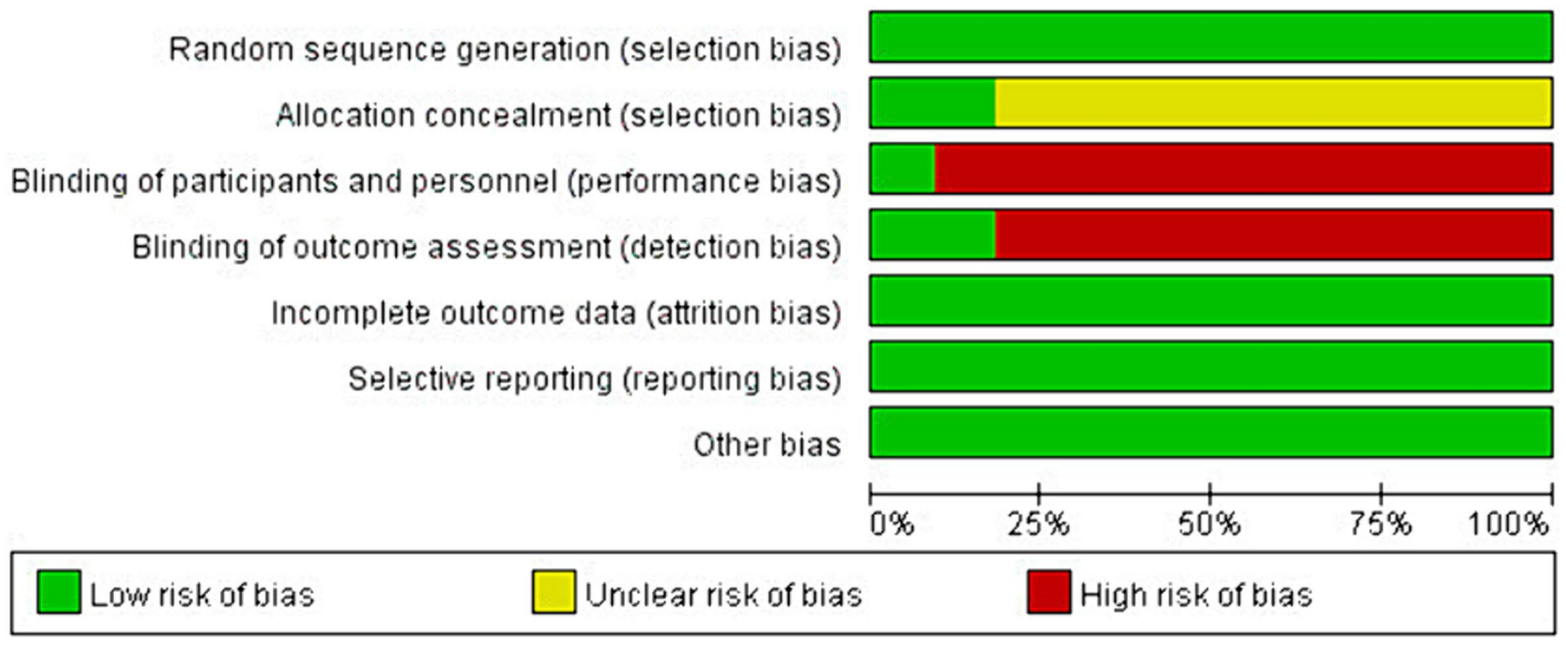
Risk of bias items as percentages across all included studies.

The Cochrane Collaboration tool was used to assess the risk of bias in RCTs for systematic review and meta-analysis. The methodological quality assessment is shown in Figures 2, 3. All studies were judged to be at low risk of bias in randomized sequence generation, completeness of results, and selective reporting. Nine studies were at uncertain risk of bias in the allocation scheme (allocation concealment) owing to the risk of bias not being specified in the article (19–22, 25, 27–29). 10 studies were judged to be at high risk of bias in the blinding of participants and personnel (19, 21–29). Nine studies at high risk of bias were judged similarly in the blinding of the outcome assessment (19, 21–27, 29). The risk of bias assessment is shown in Figures 2, 3.

### 3.4 Outcome measures

#### 3.4.1 Effect of acupuncture therapy combined with core muscle exercises on pain scores (VAS and NRS) in patients with CNLBP

Eleven RCTs (*n* = 727) involved assessing the effects of acupuncture combined with core muscle exercises on pain score outcomes (19–29). Because the NRS used by Yeung et al. (20) has the same unit of measurement as the VAS pain score, and the final post-treatment effect sizes were all mean difference values, we included them in the assessment.

**Figure 4:**
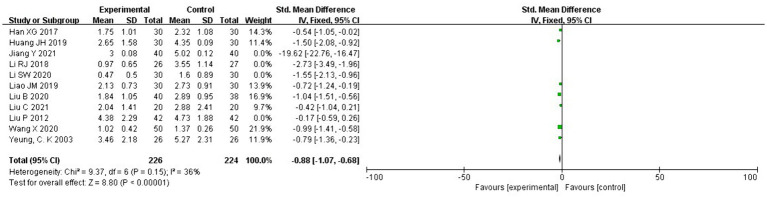
Forest plot for pain scores.

Eleven RCTs showed large heterogeneity (*I*^2^ = 95% > 50%, *p* < 0.1); therefore, we conducted a heterogeneity analysis. Sensitivity analysis was performed on the 11 RCTs in this study and revealed that Jiang Yi, Li Ruijie, Liu Chang, and Li Shuwen studies had significant heterogeneity (21, 26, 27, 29). After removing these four studies, the heterogeneity test was repeated, and the results showed that the remaining seven studies did not have heterogeneity (*I*^2^ = 36% < 50%, *p* = 0.15 > 0.1). Subsequently, the fixed effects were used to combine effect sizes, and the results showed that the difference between the two groups was statistically significant. The effect size of the remaining seven studies reached −0.88 with a 95 CI of −1.07 to −0.68 and was statistically significant (*Z* = 8.80, *p* < 0.00001). Therefore, according to the results of the fixed effects analysis, pain scores were significantly reduced in the acupuncture therapy combined with the core exercise group compared with those in the control group (Figure 4).

#### 3.4.2 Effect of acupuncture therapy combined with core muscle exercises on the clinical outcomes of patients with CNLBP

**Figure 5:**
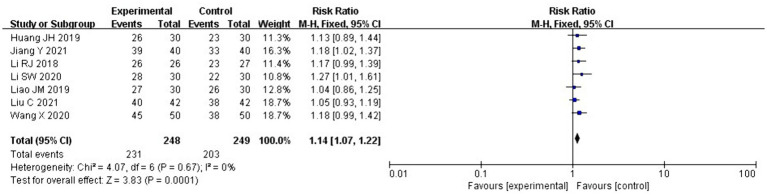
Forest plot for clinical effectiveness.

The clinical efficacy of acupuncture therapy combined with core muscle exercises for pain reduction, functional improvement, and quality of life was assessed in seven (19, 21, 24–27, 29) of the current 11 RCTs. After the heterogeneity test, *I*^2^ = 0% < 50, *p* = 0.67 > 0.1, suggesting that the heterogeneity between the selected studies was not statistically significant and that fixed effects should be selected for meta-analysis. The pooled relative risk value of the seven studies was 1.14, with a 95% CI of 1.07 to 1.22, and was statistically significant (*Z* = 3.83, *p* = 0.0001 < 0.05). Therefore, according to the fixed-effects analysis, the clinical efficacy of acupuncture combined with core exercises was more evident than that of the control group (Figure 5).

#### 3.4.3 Effect of acupuncture therapy combined with core muscle exercises on ODI in patients with CNLBP

**Figure 6:**
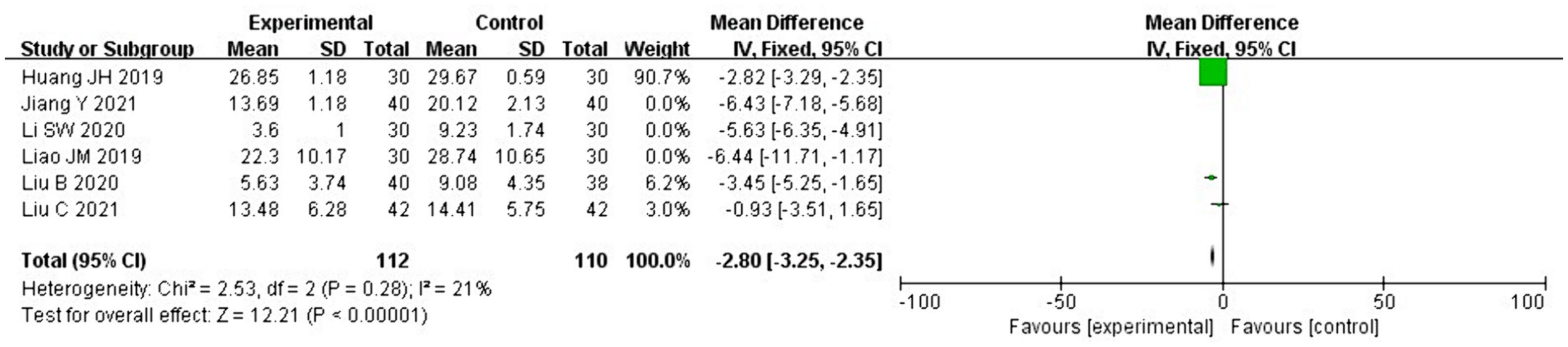
Forest plot for ODI scores.

The ODI was used to assess lumbar dysfunction, and six of the 11 RCTs in this study (21, 22, 24, 25, 27, 29) involved using the ODI scores. We deleted one study with a different calculation method (24) where heterogeneity extensively persisted (*I*^2^ = 96% > 50, *p* < 0.1), prompting a search for heterogeneity. Sensitivity analysis was performed on the current six studies, and two RCTs (27, 29) largely affected heterogeneity. After deleting these two studies, the heterogeneity test was repeated and revealed no heterogeneity in the remaining three studies (*I*^2^ = 21% < 50%; *p* = 0.28 > 0.1). The fixed effects were used to combine the effect sizes, and the results showed that the difference between the two groups was statistically significant. The remaining three study effect size reached −2.80 with a 95% CI of −3.25 to −2.35 and was statistically significant (*Z* = 12.21, *p* < 0.00001), suggesting that acupuncture therapy combined with core exercises is superior to exercise therapy alone in improving dysfunction (Figure 6).

### 3.5 Publication bias

We planned to use funnel plots to evaluate publication bias; however, the number of included trials (*n* = 11) and that of patients per trial were small (25–49). Therefore, we could not assess publication bias.

## 4 Discussion

This study primarily aimed to assess the effect of acupuncture combined with core exercise on pain and functional disability in patients with CNLBP through a systematic review and meta-analysis of RCTs. According to the results of meta-analysis, acupuncture therapy combined with core exercises can improve the pain and functional status of patients with CNLBP, and the therapeutic efficiency is significantly better than that of core exercises alone; therefore, we recommend acupuncture therapy combined with core exercises as a treatment option.

According to the results of basic and clinical studies related to the treatment of CNLBP, core stability training can activate the function of deep lumbar and abdominal muscle groups to improve lumbar spine stability (30), as well as improve pain thresholds and reduce pain intensity (31). Acupuncture therapy can inhibit inflammatory pain through peripheral, spinal and supraspinal mechanisms that activate a range of bioactive molecules containing opioid receptors, 5-hydroxytryptamine, norepinephrine and cytokines (32). Similarly, clinical trials have shown that acupuncture combined with baclofen has better clinical efficacy than baclofen alone in the treatment of CNLBP (33), and that acupuncture alone can still produce positive clinical results (34). Therefore, we believe that the combination of the two treatments may lead to better clinical outcomes.

Pain is the fifth most important vital sign in human beings (35), and the main clinical symptom of CNLBP patients is pain, and the improvement of pain is the main assessment index after acupuncture therpy combined with core muscle exercises treatment, so we used VAS score as the primary outcome index in this study. The VAS and NRS scores are pain intensity assessment scales. The NRS involves asking participants to select a number from 0 to 10 to rate their average pain intensity over the past 7 days. The VAS involves asking participants to select a point on a 0–10 cm line to represent their average pain intensity over the past 7 days, which is converted to a number. The two scores are rarely influenced by non-pain intensity in assessing a patient’s pain factors (pain or distress beliefs) and have high accuracy as pain assessment criteria (36). Yeung et al. used NRS to assess the mean pain intensity of patients with CNLBP, which is consistent with the range of VAS scores used in other studies. Moreover, according to the study, the NRS and VAS (36, 37) showed no significant differences in assessing LBP severity, and the VAS is a pain intensity measure similar to the NRS. The final post-treatment effect sizes were all mean differences, and there was no heterogeneity in the sensitivity analyses; therefore, we included the Yeung study in analyzing pain scores.

CNLBP patients also have low-back dysfunction, and the ODI is one of the most commonly used scales to assess low-back dysfunction, so we used the ODI as a secondary outcome indicator. The ODI is a research scale for assessing the functional status of patients with LBP based on the subjective evaluation of their CLBP symptoms and function. In the sensitivity analyses of the included studies, we excluded two studies with greater heterogeneity (27, 29), which involved using bladed needles and needle knives with a loosening effect. Needle knives and bladed needles originated from the ancient “nine-needle” therapy, which differs from the round needle with a pointed tip of traditional acupuncture, with a thicker diameter and a flattened and bladed tip, except for the effect of regulating qi and blood of the traditional acupuncture, which can peel off and loosen the adhesion, contracture of the tendons, and relieve the nerve and blood vessel from the pressing stimulation (38–41). The study revealed that the needle knife with loosening effect and blade needle have a better effect on improving the functional status of patients’ waist; however, we included fewer studies with smaller sample sizes, and the research data to argue the possibility of the cause of this heterogeneity are insufficient. In terms of disability assessment, a total of four scales were used as observational indicators, including ODI scores, which was involved in the meta-analysis, and The Aberdeen LBP scale (42), The Roland Morris disability questionnaire (43), and the Japanese Orthopedic Association Assessment Treatment score (23, 28). For this meta-analysis of acupuncture combined with core muscle exercise for CNLBP, the ODI scale was the most commonly used scale in the included clinical studies, and the Roland Morris disability questionnaire was less frequently used, with only one clinical study using this scale, which was insufficient to develop reliable data results and did not allow for conversion of data between scales; therefore, we did not perform a meta-analysis of the Roland Morris disability questionnaire.

In our initial statistical results, we included studies with large heterogeneity. After sensitivity analyses to exclude studies with large heterogeneity, we attempted to analyze the reasons for the statistical heterogeneity caused by the excluded studies. We considered acupuncture therapy as a treatment that involves using needles to penetrate the body to prevent and treat diseases. Therefore, we included in this retrospective analysis, different acupuncture studies that involved conventional acupuncture, electroacupuncture, needle knife (bladed needle), and fire acupuncture, with differences in the corresponding theories of these treatments, the application site, the choice of needles, and the treatment means. Therefore, the variability of the specific acupuncture therapies in the included studies may be the primary cause for statistical heterogeneity.

Our systematic review analyses were derived from comprehensive bibliographic searches of multiple databases without time constraints, followed Cochrane standards, and involved using a rigorous process and methodology. However, there are some limitations to our review. As international studies on acupuncture therapy have primarily focused on the clinical effects and mechanisms explored in acupuncture and most of the RCTs were on acupuncture versus sham acupuncture (44–51), we could not include enough relevant international studies.

We removed two English-language articles from the final studies included in the assessment. Minakawa et al. excluded people with LBP who exercised for 30 min or more at least twice a week for at least 1 year (52). The researchers of this study concluded that patients’ fear of LBP causes them to avoid physical activity. Therefore, using patient education to eliminate fear and encourage exercise can yield good results; however, older adults with exercise habits do not have associated challenges. We believe that this study improved patients’ psychological state and behavior through the provided patient education and that psychoeducational and behavioral change techniques are good facilitators for maintaining symptom improvement after LBP treatment (53). Hence, we excluded this study. Martín-Corrales et al. control group was treated with a combination of sham-dry needling based on exercise, that is, without penetrating the skin, using Park sham needles (Park Sham Device, AcuPrime, UK) on the skin to induce a tingling sensation (54). However, exploring the therapeutic mechanism of acupuncture based on meridian research theories suggests that acupuncture points are rich in sensory nerve receptors and that stimulation of acupuncture points, manually or using low currents and frequencies, reportedly works through the connection of the central nervous system to the effector organs and the integrative function of neurons in the brain (55). The research method of pseudo acupuncture, which separates the biological and psychological effects of acupuncture, does not conform with the traditional therapeutic concept of Chinese medicine, which is “unity of mind and body,” and also violates today’s “biopsychosocial” medical model; therefore, it should not be treated as a placebo in a drug trial. Sham acupuncture cannot be compared with placebo in a drug trial in a double-blind RCT; therefore, we also excluded this study.

We attempted to validate the specific effects of the duration of the exercise program and the different types of acupuncture therapies on pain, functional status, and clinical outcomes in patients with CNLBP; however, the small sample size hindered this. Thus, multi-center, large-sample trials are needed to obtain more reliable conclusions.

CLBP covers two categories, specific and non-specific, and non-specific low back pain is more common in clinical practice, accounting for about 85% (1), so this systematic review and meta-analysis only included acupuncture therapy combined with core exercise for CNLBP for relevant analysis, but specific low back pain should also attract the attention of clinicians, and we will conduct a relevant research for specific low back pain in the next study.

We concluded that acupuncture therapy combined with core exercise improved pain and function in patients with CNLBP compared with core exercise therapy alone and had good clinical efficacy. However, multicentre, large-sample trials are required to obtain more definitive conclusions.

## Data availability statement

The original contributions presented in the study are included in the article/Supplementary material, further inquiries can be directed to the corresponding author.

## Author contributions

XiL: Writing – original draft, Methodology, Investigation, Formal analysis, Data curation, Conceptualization. GZ: Writing – review & editing, Data curation, Conceptualization. HZ: Writing – review & editing, Data curation. XuL: Writing – review & editing, Data curation, Conceptualization. MW: Writing – review & editing, Data curation. SZ: Writing – review & editing, Data curation. JC: Writing – review & editing, Investigation. ZT: Writing – review & editing, Writing – original draft, Visualization, Supervision, Methodology, Funding acquisition, Formal analysis, Data curation, Conceptualization. ZH: Writing – review & editing, Data curation.
