# Supplementary material for: Clinical efficacy of acupuncture therapy combined with core muscle exercises in treating patients with chronic nonspecific low back pain: a systematic review and meta-analysis of randomized controlled trials
Source: Front Med (Lausanne). 2024 Apr 4;11:1372748. doi: 10.3389/fmed.2024.1372748 (PMC11024316; doi:10.3389/fmed.2024.1372748)
Supplement: Supplementary file 1 [file Table_1.DOCX]

Search strategy.

PUBMED

#1 ("Low Back Pain" [MeSH Terms] OR "Back Pain, Low" [Title/Abstract]OR "Back Pains, Low" [Title/Abstract]OR "Low Back Pains" [Title/Abstract]OR "Pain, Low Back" [Title/Abstract]OR "Pains, Low Back" [Title/Abstract]OR "Lumbago" [Title/Abstract]OR "Lower Back Pain" [Title/Abstract]OR "Back Pain, Lower" [Title/Abstract]OR "Back Pains, Lower" [Title/Abstract]OR "Lower Back Pains" [Title/Abstract]OR "Pain, Lower Back" [Title/Abstract]OR "Pains, Lower Back" [Title/Abstract]OR "Low Back Ache" [Title/Abstract]OR "Ache, Low Back" [Title/Abstract]OR "Aches, Low Back" [Title/Abstract]OR "Back Ache, Low" [Title/Abstract]OR "Back Aches, Low" [Title/Abstract]OR "Low Back Aches" [Title/Abstract]OR "Low Backache" [Title/Abstract]OR "Backache, Low" [Title/Abstract]OR "Backaches, Low" [Title/Abstract]OR "Low Backaches" [Title/Abstract]OR "Low Back Pain, Postural" [Title/Abstract]OR "Postural Low Back Pain" [Title/Abstract]OR "Low Back Pain, Posterior Compartment" [Title/Abstract]OR "Low Back Pain, Recurrent" [Title/Abstract]OR "Recurrent Low Back Pain" [Title/Abstract]OR "Low Back Pain, Mechanical" [Title/Abstract]OR "Mechanical Low Back Pain" [Title/Abstract])

#2 ("Acupuncture Therapy" [MeSH Terms] OR "Acupuncture Treatment" [Title/Abstract]OR "Acupuncture Treatments" [Title/Abstract]OR "Treatment, Acupuncture" [Title/Abstract]OR "Therapy, Acupuncture" [Title/Abstract]OR "Pharmacoacupuncture Treatment" [Title/Abstract]OR "Treatment, Pharmacoacupuncture" [Title/Abstract]OR "Pharmacoacupuncture Therapy" [Title/Abstract]OR "Therapy, Pharmacoacupuncture" [Title/Abstract]OR "Acupotomies" [Title/Abstract]OR "Acupotomy" [Title/Abstract])

#3 ("Exercise" [MeSH Terms] OR "Exercises" [Title/Abstract]OR "Physical Activity" [Title/Abstract]OR "Activities, Physical" [Title/Abstract] OR "Activity, Physical" [Title/Abstract]OR "Physical Activities" [Title/Abstract]OR "Exercise, Physical" [Title/Abstract]OR "Exercises, Physical" [Title/Abstract]OR "Physical Exercise" [Title/Abstract]OR "Physical Exercises" [Title/Abstract]OR "Acute Exercise" [Title/Abstract]OR "Acute Exercises" [Title/Abstract]OR "Exercise, Acute" [Title/Abstract]OR "Exercises, Acute" [Title/Abstract]OR "Exercise, Isometric" [Title/Abstract]OR "Exercises, Isometric" [Title/Abstract]OR "Isometric Exercises" [Title/Abstract]OR "Isometric Exercise" [Title/Abstract]OR "Exercise, Aerobic" [Title/Abstract]OR "Aerobic Exercise" [Title/Abstract]OR "Aerobic Exercises" [Title/Abstract]OR "Exercises, Aerobic" [Title/Abstract]OR "Exercise Training" [Title/Abstract]OR "Exercise Trainings" [Title/Abstract]OR "Training, Exercise" [Title/Abstract]OR "Trainings, Exercise" [Title/Abstract])

#4 ("randomized controlled trial" [Publication Type] OR "randomized" [Title/Abstract] OR "placebo" [Title/Abstract])

#5 "chronic" [All Fields]

#6 "non-specific" [All Fields]

#7 #1 AND #2 AND #3 AND #4 AND #5 AND #6

Web of Science

#1 TS= low* back pain* OR Back Pain*, Low* OR Pain*, Low Back OR Lumbago OR Pain*, Lower Back OR Low Back*Ache* OR Ache*, Low Back OR Back*Ache*, Low OR Postural Low Back Pain OR Recurrent Low Back Pain OR Mechanical Low Back Pain

#2 TS= Acupuncture Treatment OR Acupuncture Treatments OR Treatment, Acupuncture OR Treatment, Acupuncture OR Pharmacoacupuncture Treatment OR Treatment, Pharmacoacupuncture OR Pharmacoacupuncture Therapy OR Therapy, Pharmacoacupuncture OR Acupotomies OR Acupotomy

#3 TS=Exercise* OR Physical Activit* OR Activit* Physical OR Physical Exercise * OR Exercise* Physical OR Exercise*, Acute OR Physical Activit* OR Exercise*, Isometric OR Exercise*, Isometric OR Isometric Exercise* OR Exercise*, Physical OR Training*, Exercise OR Physical Exercise* OR Exercise Training* OR Acute Exercise* OR Exercise*, Aerobic OR Aerobic Exercise*

#4 TS=Randomi*ed controlled trial OR Random* OR Placebo

#5 TS=chronic

#6 TS=non-specific

#7 #1 AND #2 AND #3 AND #4 AND #5 AND #6

EMBASE

#1 'Back Pain, Low':ab,ti or 'Back Pains, Low':ab,ti or 'Low Back Pains':ab,ti or 'Pain, Low Back':ab,ti or 'Pains, Low Back':ab,ti or 'Lumbago':ab,ti or 'Lower Back Pain':ab,ti or 'Back Pain, Lower':ab,ti or 'Back Pains, Lower':ab,ti or 'Lower Back Pains':ab,ti or 'Pain, Lower Back':ab,ti or 'Pains, Lower Back':ab,ti or 'Low Back Ache':ab,ti or 'Ache, Low Back':ab,ti or 'Aches, Low Back':ab,ti or 'Back Ache, Low':ab,ti or 'Back Aches, Low':ab,ti or 'Low Back Aches':ab,ti or 'Backache, Low':ab,ti or 'Low Backache':ab,ti or 'Backaches, Low':ab,ti or 'Low Backaches':ab,ti or 'Low Back Pain, Postural':ab,ti or 'Postural Low Back Pain':ab,ti or 'Low Back Pain, Posterior Compartment':ab,ti or 'Low Back Pain, Recurrent':ab,ti or 'Recurrent Low Back Pain':ab,ti or 'Low Back Pain, Mechanical':ab,ti or 'Mechanical Low Back Pain':ab,ti

#2 'Acupuncture Therapy':ab,ti or 'Acupuncture Treatment':ab,ti or 'Acupuncture Treatments':ab,ti or 'Treatment, Acupuncture':ab,ti or 'Therapy, Acupuncture':ab,ti or 'Pharmacoacupuncture Treatment':ab,ti or 'Treatment, Pharmacoacupuncture':ab,ti or 'Pharmacoacupuncture Therapy':ab,ti or 'Therapy, Pharmacoacupuncture':ab,ti or 'Acupotomies':ab,ti or 'Acupotomy':ab,ti

#3 'Exercises':ab,ti or 'Physical Activity':ab,ti or 'Activities, Physical':ab,ti or 'Activity, Physical':ab,ti or 'Physical Activities':ab,ti or 'Exercise, Physical':ab,ti or 'Exercises, Physical':ab,ti or 'Physical Exercise':ab,ti or 'Physical Exercises':ab,ti or 'Acute Exercise':ab,ti or 'Acute Exercises':ab,ti or 'Exercise, Acute':ab,ti or 'Exercises, Acute':ab,ti or 'Exercise, Isometric':ab,ti or 'Exercises, Isometric':ab,ti or 'Isometric Exercises':ab,ti or 'Isometric Exercise':ab,ti or 'Low Back Pains':ab,ti or 'Aerobic Exercise':ab,ti or 'Aerobic Exercises':ab,ti or 'Exercises, Aerobic':ab,ti or 'Exercise Training':ab,ti or 'Exercise Trainings':ab,ti or 'Training, Exercise':ab,ti or 'Trainings, Exercise':ab,ti

#4 'Randomized controlled trial':ab,ti or 'Randomized':ab,ti or 'Placebo':ab,ti

#5 'chronic':ab,ti or

#6 'non-specific ':ab,ti or

#7 #1 AND #2 AND #3 AND #4 AND #5 AND #6

Cochrane Library

#1 MeSH descriptor: [Low Back Pain] explode all trees

#2 (Back Pain, Low):ti,ab,kw OR (Back Pains, Low):ti,ab,kw OR (Low Back Pains):ti,ab,kw OR (Pain, Low Back):ti,ab,kw OR (Pains, Low Back):ti,ab,kw OR (Lumbago):ti,ab,kw OR (Lower Back Pain):ti,ab,kw OR (Back Pain, Lower):ti,ab,kw OR (Back Pains, Lower):ti,ab,kw OR (Lower Back Pains):ti,ab,kw OR (Pain, Lower Back):ti,ab,kw OR (Pains, Lower Back):ti,ab,kw OR (Low Back Ache):ti,ab,kw OR (Ache, Low Back):ti,ab,kw OR (Aches, Low Back):ti,ab,kw OR (Back Ache, Low):ti,ab,kw OR (Back Aches, Low):ti,ab,kw OR (Low Back Aches):ti,ab,kw OR (Low Backache):ti,ab,kw OR (Backache, Low):ti,ab,kw OR (Backaches, Low):ti,ab,kw OR (Low Backaches):ti,ab,kw OR (Low Back Pain, Postural):ti,ab,kw OR (Postural Low Back Pain):ti,ab,kw OR (Low Back Pain, Posterior Compartment):ti,ab,kw OR (Low Back Pain, Recurrent):ti,ab,kw OR (Recurrent Low Back Pain):ti,ab,kw OR (Low Back Pain, Mechanical):ti,ab,kw OR (Mechanical Low Back Pain):ti,ab,kw

#3 #1 OR #2

#4 (non-specific):ti,ab,kw

#5 (chronic):ti,ab,kw

#6 #3 AND #4 AND #5

#7 (Acupuncture Treatment):ti,ab,kw OR (Acupuncture Treatments):ti,ab,kw OR (Treatment,Acupuncture):ti,ab,kw OR (Therapy,Acupuncture):ti,ab,kw OR (Pharmacoacupuncture Treatment):ti,ab,kw OR (Treatment,Pharmacoacupuncture):ti,ab,kw OR (Pharmacoacupuncture Therapy):ti,ab,kw OR (Therapy,Pharmacoacupuncture):ti,ab,kw OR (Acupotomies):ti,ab,kw OR (Acupotomy):ti,ab,kw

#8 MeSH descriptor: [Acupuncture Therapy] explode all trees

#9 #7 OR #8

#10 #6 AND #9

#11 MeSH descriptor: [Exercise] explode all trees 38838

#12 (Exercise):ti,ab,kw OR (Exercises):ti,ab,kw OR (Physical Activity):ti,ab,kw OR (Activities, Physical):ti,ab,kw OR (Activity, Physical):ti,ab,kw OR (Physical Activities):ti,ab,kw OR (Exercise, Physical):ti,ab,kw OR (Exercises, Physical):ti,ab,kw OR (Physical Exercise):ti,ab,kw OR (Physical Exercises):ti,ab,kw OR (Acute Exercise):ti,ab,kw OR (Acute Exercises):ti,ab,kw OR (Exercise, Acute):ti,ab,kw OR (Exercises, Acute):ti,ab,kw OR (Exercise, Isometric):ti,ab,kw OR (Exercises, Isometric):ti,ab,kw OR (Isometric Exercises):ti,ab,kw OR (Isometric Exercise):ti,ab,kw OR (Exercise, Aerobic):ti,ab,kw OR (Aerobic Exercise):ti,ab,kw OR (Aerobic Exercises):ti,ab,kw OR (Exercises, Aerobic):ti,ab,kw OR (Exercise Training):ti,ab,kw OR (Exercise Trainings):ti,ab,kw OR (Training, Exercise):ti,ab,kw OR (Trainings, Exercise):ti,ab,kw

#13 #11 OR #12

#14 #10 AND #13

CNKI

(SU=*'* chronic non-specific low back pain *' + '* chronic low back pain *'*) AND (SU=*'* acupuncture therapy*' + '* needle *'*) AND (SU=*'* exercise *'*) AND (AB=*'* randomized controlled trial *'+ '* randomized *'*)

Wanfang

(SU=*'* chronic non-specific low back pain *' + '* chronic low back pain *'*) AND (SU=*'* acupuncture therapy*' + '* needle *'*) AND (SU=*'* exercise *'*) AND (SU=*'* randomized controlled trial *'+ '* randomized *'*)

Chinese Biomedical Literature

#1 ("chronic non-specific low back pain " [All Fields] OR"chronic low back pain " [All Fields]) AND “low back pain”[MeSH Terms]

#2 ("acupuncture therapy " [All Fields] OR"needle " [All Fields] ) AND “acupuncture”[MeSH Terms]

#3 "exercise " [All Fields] )

#4 "randomized controlled trial" [All Fields]*+* "randomized" [All Fields]
